# Evaluation of Prosthodontic Status, Treatment Needs, Nutritional Risks, and Oral Health-Related Quality of Life in Rural and Urban Older Adults of Pune District: A Cross-Sectional Study

**DOI:** 10.7759/cureus.111321

**Published:** 2026-06-22

**Authors:** Chetan Modgi, Sasmita Dalai, Anjali Sharma, Asmita Ghosh, Ikroop Gill, Amanurrahman Zubair Ahemad, Ekta Choudhary, Tarun Kumar Singh

**Affiliations:** 1 Department of Prosthodontics, Crown and Bridge, Government Dental College, Silchar, IND; 2 Department of Dentistry, All India Institute of Medical Sciences, Bathinda, IND; 3 Department of Conservative Dentistry and Endodontics, Teerthanker Mahaveer Dental College and Research Centre, Moradabad, IND; 4 Department of Conservative Dentistry and Endodontics, Sharda School of Dental Sciences, Greater Noida, IND

**Keywords:** geriatrics, nutrition assessment, oral health, prosthodontics, quality of life

## Abstract

Introduction: Oral health-related quality of life (OHRQoL) and nutritional status are important determinants of overall well-being among older adults. Compromised prosthetic status and unmet prosthetic treatment needs may adversely influence mastication, nutrition, and psychosocial health, particularly among older adults residing in underserved regions. This study aimed to evaluate the prosthodontic status, prosthetic treatment needs, nutritional status, and oral health-related quality of life among geriatric individuals residing in rural and urban areas of Pune district, Maharashtra, India.

Materials and methods: This analytical cross-sectional study included 1,500 geriatric participants aged ≥ 60 years, including 750 rural and 750 urban participants. The prosthodontic status and prosthetic treatment needs were assessed. Oral health-related quality of life was evaluated using the Oral Health Impact Profile-14 (OHIP-14) questionnaire, and nutritional status was assessed using the Mini Nutritional Assessment-Short Form (MNA-SF) questionnaire. Statistical analyses were performed using the Statistical Package for the Social Sciences (SPSS), version 23.0 (IBM Corp., Armonk, NY). Descriptive statistics, the Chi-square test, and the independent samples t-test were applied. Statistical significance was set at p < 0.05.

Results: The majority of participants were aged 60-69 years, accounting for 1,350 (90.0%) of the study population, while males comprised 1,035 (69.0%) of the participants. Rural participants had significantly greater prosthetic treatment needs than urban participants. Full prosthetic rehabilitation in the upper jaw was required by 357 (47.6%) rural participants compared with 50 (6.7%) urban participants. Full prosthetic rehabilitation of the lower jaw was required by 459 (61.2%) rural participants compared with 126 (16.8%) urban participants. Rural participants demonstrated significantly higher mean OHIP-14 scores (14.55 ± 3.39) than urban participants (10.33 ± 2.87), indicating a poorer oral health-related quality of life. Urban participants exhibited significantly higher mean MNA-SF scores (10.75 ± 1.03) than rural participants (10.04 ± 0.95), suggesting a comparatively better nutritional status (p < 0.001).

Conclusion: Rural older adults exhibited greater unmet prosthetic needs, poorer nutritional status, and compromised oral health-related quality of life compared with urban participants. An association was observed between prosthodontic status, nutritional status, and oral health-related quality of life.

## Introduction

Health is defined by the World Health Organization as a state of complete physical, mental, and social well-being, not merely the absence of disease. This multidimensional concept of health has led to an increasing emphasis on quality of life and functional well-being in healthcare research and practice [1]. In recent years, oral health has emerged as an essential component of general health because oral diseases significantly influence mastication, speech, appearance, nutrition, and psychosocial well-being [2]. Therefore, oral health-related quality of life (OHRQoL) has gained considerable importance in understanding the impact of oral conditions on daily functioning and overall life satisfaction [3].

Global increases in life expectancy have resulted in a growing geriatric population. Older adults are more susceptible to chronic systemic diseases, age-related degenerative changes, tooth loss, edentulism, and impaired oral function. These conditions adversely affect nutritional intake and may compromise systemic health [4]. Prosthodontic rehabilitation plays a vital role in restoring oral function, improving esthetics, enhancing nutritional status, and maintaining psychological well-being among older adults, particularly those with systemic diseases, such as type 2 diabetes mellitus [5].

Various indices such as the Oral Health Impact Profile-14 (OHIP-14) and Mini Nutritional Assessment-Short Form (MNA-SF) are widely used to evaluate OHRQoL and nutritional status in geriatric populations [6,7]. Although prosthodontic status, treatment needs, nutritional status, and OHRQoL have been investigated separately in older adults, limited evidence is available regarding their combined assessment, particularly among rural and urban geriatric populations in India. Therefore, the present cross-sectional study was undertaken to evaluate the association between sociodemographic and nutritional factors and prosthodontic status, treatment needs, and OHRQoL among older adults residing in Pune district, Maharashtra, India. The objectives of the study were to assess prosthodontic status and prosthetic treatment needs and to evaluate OHRQoL and nutritional status among older adults residing in rural and urban areas of Pune district, Maharashtra.

## Materials and methods

Study design, setting, ethical approval

The present analytical cross-sectional study was conducted among the older adults residing in rural and urban areas of the Pune district, Maharashtra, India. The study was carried out in the Department of Prosthodontics from February 2018 to March 2020 after obtaining ethical approval from the Institutional Ethics Committee of VSPM's Dental College and Research Center, Nagpur, India (Ph'D/IEC/VSPMDCRC/02/2018, dated 08/01/2018). Written informed consent was obtained from all participants prior to the commencement of the study, after explaining the nature and purpose of the study in their understandable language. Confidentiality of participant information was maintained throughout the study.

Study population

The study population comprised community-dwelling older adults aged 60 years and above residing in rural and urban areas of the Pune district, Maharashtra, India. Participants who fulfilled the inclusion criteria and were willing to provide informed consent were enrolled in this study.

Sample size estimation and sampling technique

The sample size was estimated based on the prevalence of 80% prosthetic treatment needs among older adults as reported in the literature [8]. Sample size calculation was performed using OpenEpi software, version 3.01 (Open Source Epidemiologic Statistics for Public Health, Atlanta, Georgia, USA). Using a confidence level of 95%, an absolute precision of 5%, and a design effect of 2 for cluster sampling, the minimum required sample size was calculated to be 690 participants per group. To compensate for potential nonresponse and incomplete data, the sample size was increased to 750 participants from each rural and urban area, resulting in a total sample size of 1,500 participants.

A cluster sampling technique was employed for participant selection. Rural and urban regions were divided into predefined clusters, which were selected according to the sampling framework. Eligible participants were recruited from the selected clusters until the required sample size was achieved. Participants aged ≥ 60 years who were present at the time of examination and willing to provide informed consent were included in the study. Participants who were bedridden, medically compromised, unable to undergo examination, or unwilling to participate were excluded from the study.

Data collection procedure

Data were collected through direct clinical examinations and face-to-face interviews, using a structured assessment format. Sociodemographic details, including age, sex, education, living conditions, dietary habits, systemic diseases, and dependency status, were recorded. Prosthodontic status and prosthetic treatment needs were assessed according to the World Health Organization criteria for geriatric prosthodontic treatment needs [9].

Assessment of oral health-related quality of life and nutritional status

Oral health-related quality of life was assessed using the OHIP-14 questionnaire. The OHIP-14 questionnaire evaluates the functional, psychological, and social impact of oral health conditions on an individual’s quality of life. The questionnaire included 14 items distributed across seven domains, and responses were recorded according to standardized scoring criteria [6]. Nutritional status was assessed using the MNA-SF questionnaire. The MNA-SF includes the evaluation of food intake, weight loss, mobility, psychological stress, neuropsychological problems, and body mass index to determine nutritional risk among geriatric participants [7]. The functional status of existing prostheses was assessed during clinical examination. A prosthesis was considered functional when it was present, adequately retained and stable, and capable of providing satisfactory masticatory function without causing discomfort. Prostheses exhibiting poor retention, instability, fracture, excessive wear, improper fit, or those deemed inadequate for effective mastication were classified as non-functional.

Training and calibration

Prior to the commencement of the study, the examiner underwent training and calibration to ensure consistency and reliability in the clinical examination and questionnaire administration. Standard infection control measures and examination protocols were followed throughout the study.

Data management and statistical analysis

The collected data were entered into Microsoft Excel 2013 (Microsoft Corporation, Redmond, Washington, USA) and analyzed using the Statistical Package for the Social Sciences version 23.0 (IBM Corporation, Armonk, New York, USA). Descriptive statistics are expressed as frequencies, percentages, means, and standard deviations. The normality of the OHIP-14 and MNA-SF scores was assessed using the Shapiro-Wilk test. The Chi-square test was applied for the comparison of categorical variables, while the independent samples t-test was used for the comparison of mean scores between rural and urban participants. Statistical significance was set at p < 0.05.

## Results

Table 1 shows the demographic characteristics of the rural and urban participants. Most participants in both groups were aged 60-69 years, male, and independent in daily activities, with no significant differences between the groups (p > 0.05). Significant differences were observed in living condition, education, diet, and systemic disease status (p < 0.05). Rural participants more commonly lived alone and had a higher prevalence of systemic diseases, whereas urban participants showed higher educational attainment.

**Table 1:** Demographic characteristics of study participants in rural and urban groups (n = 750 each) Data are presented as frequency and percentage, intergroup comparisons were performed using the Chi-square test, *p-value < 0.05 was considered statistically significant, χ²: Chi-square value, df: degrees of freedom.

Variable	Category	Rural (n = 750) n (%)	Urban (n = 750) n (%)	χ² value (df)	p-value
Age group (years)	60-69	670 (89.3)	680 (90.7)	0.74 (1)	0.389
	70-79	80 (10.7)	70 (9.3)		
Sex	Male	525 (70.0)	510 (68.0)	0.70 (1)	0.402
	Female	225 (30.0)	240 (32.0)		
Level of dependency	Independent	700 (93.3)	690 (92.0)	0.98 (1)	0.322
	Dependent	50 (6.7)	60 (8.0)		
Living Condition	Living alone	41 (5.5)	10 (1.3)	18.27 (1)	< 0.001*
	Not living alone	709 (94.5)	740 (98.7)		
Education	Primary	487 (64.9)	466 (62.1)	48.08 (3)	< 0.001*
	Secondary	121 (16.1)	55 (7.3)		
	Graduation	76 (10.1)	142 (18.9)		
	Post-graduation	66 (8.8)	87 (11.6)		
Diet	Vegetarian	64 (8.5)	42 (5.6)	4.48 (1)	0.034*
	Mixed	686 (91.5)	708 (94.4)		
Systemic disease status	Present	706 (94.1)	545 (72.6)	123.27 (1)	< 0.001*
	Absent	44 (5.9)	205 (27.4)		

Table 2 shows a comparison of prosthetic and functional status between rural and urban participants. In the upper jaw, rural participants had a higher prevalence of removable upper dentures (32.4% vs. 15.4%), whereas urban participants demonstrated a higher prevalence of complete upper dentures (50.6% vs. 31.2%). Upper fixed multiple partial dentures among urban participants constituted 218 (29.0%), whereas among rural participants, they constituted 40 (5.3%). In the lower jaw, rural participants without prostheses constituted 332 (44.3%), whereas urban participants with lower fixed multiple partial dentures constituted 360 (48.0%). Functional upper prostheses among urban participants numbered 652 (86.9%), whereas the corresponding rural participants numbered 136 (18.1%). Among rural participants, 421 (56.1%) had non-functional upper prostheses, whereas urban participants constituted 62 (8.2%). Functional lower prostheses among urban participants constituted 365 (48.6%), whereas rural participants constituted 85 (11.3%). These differences were statistically significant (p < 0.001).

**Table 2:** Comparison of prosthetic status and functional status between rural and urban geriatric participants. Data are presented as frequency and percentage, intergroup comparison was performed using the Chi-square test, *p-value of less than 0.05 was considered statistically significant, df: degrees of freedom.

Variable	Category	Rural (n = 750) n (%)	Urban (n = 750) n (%)	χ² value (df)	p-value
Prosthetic status (Upper jaw)	No prosthesis	193 (25.7)	36 (4.8)	350.09 (4)	< 0.001*
	Upper complete denture	234 (31.2)	380 (50.6)		
	Upper removable denture	243 (32.4)	116 (15.4)		
	Upper multiple fixed partial denture	40 (5.3)	218 (29.0)		
	Upper six-unit fixed partial denture	40 (5.3)	0 (0.0)		
Prosthetic status (Lower jaw)	No prosthesis	332 (44.3)	36 (4.8)	513.58 (3)	< 0.001*
	Lower complete denture	179 (23.9)	224 (29.8)		
	Lower removable partial denture	199 (26.5)	130 (17.3)		
	Lower fixed multiple partial denture	40 (5.3)	360 (48.0)		
Functional status (Upper jaw)	No prosthesis	193 (25.7)	36 (4.8)	712.36 (2)	< 0.001*
	Yes (functional)	136 (18.1)	652 (86.9)		
	No (non-functional)	421 (56.1)	62 (8.2)		
Functional status (Lower jaw)	No prosthesis	332 (44.3)	36 (4.8)	412.68 (2)	< 0.001*
	Yes (functional)	85 (11.3)	365 (48.6)		
	No (non-functional)	333 (44.4)	349 (46.5)		

Table 3 presents a comparison of prosthetic treatment needs between rural and urban participants. In the upper jaw, full prosthetic rehabilitation was required by 357 (47.6%) rural participants compared with 50 (6.7%) urban participants. No prosthetic treatment was needed among 690 (92.0%) urban participants and 137 (18.3%) rural participants. Multiunit prosthetic treatment was required by 168 (22.4%) rural participants compared with 10 (1.3%) urban participants. In the lower jaw, full prosthetic rehabilitation was required by 459 (61.2%) rural participants and 126 (16.8%) urban participants. No prosthetic treatment was needed among 401 (53.4%) urban participants compared with 88 (11.7%) rural participants. The intergroup differences in prosthetic treatment needs were statistically significant (p < 0.001).

**Table 3:** Comparison of prosthetic treatment needs between rural and urban geriatric participants. Data are presented as frequency and percentage, intergroup comparison was performed using the Chi-square test, *p-value of less than 0.05 was considered statistically significant, df: degrees of freedom.

Variable	Category	Rural (n = 750) n (%)	Urban (n = 750) n (%)	χ² value (df)	p-value
Prosthetic needs (upper Jaw)	No prosthesis needed	137 (18.3)	690 (92.0)	1109.82 (3)	< 0.001*
	Multi-unit prosthesis needed	168 (22.4)	10 (1.3)		
	Combination of one and/or multi-unit	88 (11.7)	0 (0.0)		
	Full prosthesis needed	357 (47.6)	50 (6.7)		
Prosthetic needs (lower Jaw)	No prosthesis needed	88 (11.7)	401 (53.4)	440.18 (3)	< 0.001*
	Multi-unit prosthesis needed	117 (15.6)	42 (5.6)		
	Combination of one and/or multi-unit	86 (11.5)	181 (24.1)		
	Full prosthesis needed	459 (61.2)	126 (16.8)		

Table 4 shows a comparison of OHIP-14 and MNA-SF scores between rural and urban participants. Rural participants demonstrated a significantly higher mean OHIP-14 score (14.55 ± 3.39) than urban participants (10.33 ± 2.87), indicating poorer oral health-related quality of life among rural participants. Conversely, urban participants demonstrated a significantly higher mean MNA-SF score (10.75 ± 1.03) than rural participants (10.04 ± 0.95), indicating comparatively better nutritional status among urban participants. These differences were statistically significant (p < 0.001).

**Table 4:** Comparison of oral health-related quality of life and nutritional status scores between rural and urban geriatric participants. Data are presented as mean ± standard deviation, intergroup comparison was performed using the independent samples t-test, *p-value of less than 0.05 was considered statistically significant, OHIP-14: Oral Health Impact Profile-14, MNA-SF: Mini Nutritional Assessment–Short Form, SD: Standard deviation, df: degrees of freedom.

Variable	Rural (n = 750) Mean ± SD	Urban (n = 750) Mean ± SD	Mean difference	t value	df	p-value
OHIP score	14.55 ± 3.39	10.33 ± 2.87	4.22	26.03	1498	< 0.001*
MNA-SF score	10.04 ± 0.95	10.75 ± 1.03	-0.71	-13.88	1498	< 0.001*

## Discussion

The present cross-sectional study was conducted to evaluate prosthodontic status, treatment needs, OHRQoL, and nutritional status among older adults residing in rural and urban areas of the Pune district, Maharashtra, India. In the present study, the majority of participants belonged to the age group of 60-69 years, with males constituting a greater proportion of the study population. Similar demographic trends were reported by Deogade et al. [8], who observed a higher proportion of younger elderly individuals seeking prosthodontic care in institutionalized populations. The predominance of independent older adults observed in the present study may be attributed to the inclusion of community-dwelling older adults rather than institutionalized or bedridden individuals.

The present study demonstrated a significantly higher prevalence of missing prostheses and non-functional prostheses among rural participants than among urban participants. Rural participants exhibited greater dependence on removable prostheses, whereas urban participants demonstrated a greater prevalence of complete dentures and fixed prostheses [10]. These findings are in accordance with the observations of Srisilapanan et al. [11], who reported unmet prosthodontic treatment needs among elderly populations due to limited accessibility, socioeconomic constraints, and reduced awareness regarding oral rehabilitation. The greater prevalence of fixed prostheses among the urban participants in the present study may be related to improved accessibility to dental services, higher literacy levels, and better financial resources in urban settings.

The present study also indicated that rural participants had significantly greater prosthetic treatment needs in both the upper and lower jaws than urban participants. Full prosthetic rehabilitation is predominantly required for elderly rural individuals. Similar findings were reported by Deogade et al. [8], who demonstrated a substantial burden of unmet prosthetic treatment needs among the elderly population in India. The high prevalence of prosthetic needs among rural participants may be associated with reduced oral healthcare utilization, poor awareness of preventive dental care, and limited availability of prosthodontic services in rural regions.

OHRQoL was significantly poorer among rural participants, as evidenced by their higher OHIP-14 scores. The findings of the present study are consistent with those reported by Ikebe et al. [12], who demonstrated that oral condition, tooth loss, and compromised prosthetic status negatively influence daily activities, psychological well-being, and social interactions among elderly individuals. Poor oral function and compromised mastication may contribute to reduced confidence, social embarrassment, and diminished quality of life among geriatric populations [13].

The findings of our study further indicated significantly lower MNA-SF scores among rural participants, indicating poorer nutritional status compared to urban participants. These findings suggest an association between oral health and nutritional status among older adults. Similar observations were reported by Sheiham et al. [14] and Sahyoun et al. [15], who found that compromised dentition and reduced posterior occlusal support were associated with lower nutrient intake and poorer dietary quality in older adults. Elderly individuals with missing teeth or non-functional prostheses often experience difficulty in mastication, thereby limiting the consumption of fibrous and nutrient-rich foods and ultimately predisposing them to malnutrition [16].

The findings highlight the importance of comprehensive geriatric oral health care, given the greater prosthetic treatment needs, poorer nutritional status, and compromised oral health-related quality of life observed among rural participants. Prosthodontic rehabilitation not only improves mastication and esthetics but also contributes significantly to psychological well-being and nutritional adequacy [17]. Early identification of prosthetic needs and timely oral rehabilitation may improve both oral and systemic health outcomes in elderly individuals.

Recent concepts such as oral frailty and functional dentition provide additional insight into the relationship between prosthodontic status, nutrition, and quality of life among older adults. Oral frailty refers to the age-related decline in oral function, including reduced masticatory efficiency, swallowing ability, and oral muscle strength, which may adversely affect dietary intake and overall health. Similarly, the maintenance of functional dentition through natural teeth or adequately functioning prostheses is considered important for preserving oral function and supporting nutritional well-being [18]. Therefore, timely prosthodontic rehabilitation may help maintain oral function and improve quality of life in aging populations.

The clinical implications of the present study emphasize the need for community-based geriatric oral healthcare programs, particularly in rural regions. Routine prosthodontic screening, oral health education, nutritional counseling, and affordable prosthetic rehabilitation services should be integrated into public health programs for the elderly. These findings further support the need for interdisciplinary collaboration between prosthodontists, nutritionists, and primary healthcare professionals to improve the overall quality of life among geriatric individuals.

Despite its strengths, the present study had certain limitations. Because this was a cross-sectional study, causal relationships between prosthodontic status, nutritional status, and oral health-related quality of life could not be established. The study was conducted in a single district of Maharashtra, which may limit the generalizability of the findings to other populations. Self-reported responses obtained through the OHIP-14 and MNA-SF questionnaires may have introduced response bias. In addition, factors such as socioeconomic status, oral hygiene practices, healthcare access, oral health awareness, and other behavioral determinants that may influence prosthodontic treatment utilization and oral health outcomes were not evaluated in detail. Furthermore, multivariable analyses were not performed; therefore, the potential influence of confounding factors on the observed associations could not be fully assessed. Future longitudinal and multicentric studies incorporating these variables are recommended to provide a more comprehensive understanding of the factors associated with prosthodontic status, nutritional health, and oral health-related quality of life among geriatric populations.

## Conclusions

The present study demonstrated significant differences in prosthodontic status, treatment needs, nutritional status, and OHRQoL between rural and urban geriatric populations. Rural participants exhibited greater unmet prosthetic needs, poorer nutritional status, and compromised OHRQoL compared with urban participants. These findings emphasize the importance of addressing prosthodontic treatment needs and promoting oral health among elderly individuals, particularly in rural communities. Early identification of prosthetic needs and implementation of accessible community-based prosthodontic care programs may contribute to improved quality of life and nutritional well-being among the geriatric population.
